# Aspirin and lung cancer in women

**DOI:** 10.1038/sj.bjc.6600370

**Published:** 2002-07-15

**Authors:** A Akhmedkhanov, P Toniolo, A Zeleniuch-Jacquotte, K L Koenig, R E Shore

**Affiliations:** Department of Obstetrics and Gynecology, New York University School of Medicine, 550 First Avenue, NBV-9E2, New York, NY 10016, USA; Department of Environmental Medicine, New York University School of Medicine, 650 First Avenue, New York, NY 10016, USA

**Keywords:** aspirin, lung neoplasms, female, case–control study, epidemiology

## Abstract

The association between aspirin use and lung cancer risk in women was examined in a case–control study nested in the New York University Women's Health Study, a large cohort in New York. Case subjects were all the 81 incident lung cancer cases who had provided information about aspirin use at enrollment and during the 1994–1996 follow up. Ten controls per case were randomly selected from among study participants who matched a case by age, menopausal status, and dates of enrollment and follow-up. Relative to no aspirin use, the odds ratio for lung cancer (all histological sub-types combined) among subjects who reported aspirin use three or more times per week for at least 6 months was 0.66 (95% confidence interval 0.34–1.28), after adjustment for smoking and education. A stronger inverse association was observed in analyses restricted to non-small cell lung cancer (adjusted odds ratio 0.39, 95% confidence interval 0.16–0.96). These results suggest that regular aspirin use might be inversely associated with risk of lung cancer in women, particularly the non-small cell sub-type.

*British Journal of Cancer* (2002) **87**, 49–53. doi:10.1038/sj.bjc.6600370 www.bjcancer.com

© 2002 Cancer Research UK

## 

A role for nonsteroidal anti-inflammatory drugs (NSAIDs) in preventing lung carcinogenesis is suggested by animal models, in which aspirin and other NSAIDs inhibited the formation of chemically-induced lung tumours (Jalbert and Castonguay, 1992; Castonguay *et al*, 1998; Rioux and Castonguay, 1998).

The relation between NSAIDs use and lung cancer has been examined in at least six epidemiological studies published to date. In a trial of aspirin among British physicians, there were fewer lung cancer deaths among aspirin users compared with nonusers (Peto *et al*, 1988). In two prospective studies, no overall association was observed between aspirin use and lung cancer, although when stratified by gender, regular aspirin use appeared to be inversely associated with lung cancer incidence (Paganini-Hill *et al*, 1989) and mortality (Thun *et al*, 1993) in women. In a prospective study based on the first National Health and Nutrition Examination Survey, self-reported use of aspirin during the 30-day period preceding a subject's recruitment into the cohort was associated with a significant reduction in lung cancer incidence compared with nonuse (Schreinemachers and Everson, 1994). Two case–control studies found no significant associations between aspirin use and risk of lung cancer (Rosenberg, 1995; Langman *et al*, 2000).

To assess further the potential effect of aspirin use on lung cancer risk, we analysed the data in a case–control study of lung cancer nested in the New York University (NYU) Women's Health Study cohort.

## MATERIALS AND METHODS

### Study population

The NYU Women's Health Study is a long-term prospective cohort study that has been described in detail elsewhere (Toniolo *et al*, 1991, 1995). Briefly, between March 1985 and June 1991, 14 275 mostly Caucasian women between ages of 31 and 70 years were enrolled at a mammography screening clinic in New York City. Women, who in the preceding 6 months had neither used hormonal medications nor been pregnant, were eligible for enrollment. At the time of their recruitment into the cohort, study subjects answered a self-administered questionnaire focusing on demographic, anthropometric, reproductive and dietary data, medical history and medication use. Approximately every 2 years, follow-up questionnaires were mailed to all cohort members to update the information on potential risk factors and to identify newly diagnosed cases of cancer and other medical conditions. Written informed consent was obtained from all subjects. The study was reviewed and approved annually by the Institutional Board of Research Associates of NYU School of Medicine.

### Ascertainment of exposure

Information on individual use of aspirin and other NSAIDs was obtained at baseline and in a more detailed format in the 1994–1996 follow-up questionnaire. The baseline questionnaire (1985–1991) included the following question: *In the last 4 weeks, have you taken pain killers such as Aspirin, Motrin, Naprosyn, etc.?* For subjects who responded positively, specific information, including the name of the drug and when last taken, was collected. Subjects who reported use of any drug recorded in the Physicians' Desk Reference® 2000 edition as containing acetylsalicylic acid, were classified as exposed to aspirin at baseline. In the 1994–1996 follow-up questionnaire, additional information on aspirin use was collected by means of the following question: *Have you taken Aspirin three or more times per week for a period of 6 months or longer?* For those who answered positively, we queried the age when aspirin use started, the age when it stopped and total duration of treatment.

### Identification of cases

Lung cancer cases were identified primarily through active follow-up either during annual visits for mammographic screening (up to 1991) or through follow-up questionnaires mailed to each cohort member approximately every 2 years. The active follow-up was supplemented with regular record linkages with the statewide Tumor Registries of New York, New Jersey, Connecticut and Florida, and with the US National Death Index. For each reported case of lung cancer, we requested individuals, or their next of kin, for permission to obtain a copy of the pertinent medical and pathology reports.

### Case–control study

As of 1 July, 2000, after a median follow-up of 12 years (137 700 person-years), 144 women with incident lung cancer were identified. Pathological confirmation of these lung cancer cases was achieved either through medical record review (96 cases) or through linkages with Tumor Registries (42 cases). For six reported cases of lung cancer the pathology reports were not available at the time of the study. Because of the high concordance between reported and independently confirmed lung cancer in our study, these cases were retained in the final analyses.

Ten controls per case were selected at random among cohort members who were alive and free of cancer at the time of the diagnosis of a given case and who matched the case on age at enrollment (±6 months), menopausal status at enrollment, date of enrollment (±6 months) and date of response to the 1994–1996 follow-up questionnaire (±6 months). As a result, 1584 women were eligible for the case–control study, including 144 cases of lung cancer and 1440 individually matched controls.

Of the 1584 eligible subjects, 1507 (81 lung cancer cases, 1426 controls) had replied to questions on aspirin use in the 1994–1996 follow-up questionnaire. Among the latter, 618 were controls from matched sets containing non-respondent cases and were dropped. As a result, 81 lung cancer cases and 808 individually-matched control subjects were considered for study. Of the 81 lung cancer cases, 51 had answered the questions about long-term aspirin use prior to their diagnosis and 30 cases after diagnosis.

### Data analysis

Information on potential confounders, such as age, menopausal status, and body mass index (BMI; weight in kilograms divided by height in meters squared), was obtained from the baseline questionnaire. Detailed information on race/ethnicity, educational status and smoking history was collected using subsequent follow-up questionnaires. Information on aspirin use was obtained from the baseline and the 1994–1996 follow-up questionnaires, as described above.

Within each matched set, aspirin exposure was discounted for both cases and corresponding controls if it occurred after the date of diagnosis of the case. In addition, we also discounted aspirin exposures within 1 year of the date of diagnosis by censoring aspirin exposures occurring after the index date (date of diagnosis – 1 year) for both cases and their matched controls. A lag period of 1 year was chosen to prevent the possibility of protopathic bias, i.e., aspirin use by case subjects might have been the result of early symptoms of an undiagnosed disease and therefore be a consequence of, rather than a risk factor for, disease.

Conditional logistic regression analysis appropriate for matched case–control data was used to assess the association between aspirin use and lung cancer. Potential confounders were selected with a backward elimination strategy, decided upon *a priori* to avoid overadjustment (Day *et al*, 1980). This approach resulted in two statistically significant determinants of lung cancer risk: smoking history and educational status (*P*<0.001 and 0.03, respectively). Adjustment for potential confounders for lung cancer was performed by means of inclusion of variables for smoking (never, past, current) and education (no college, attended college, attended graduate school) in the multivariate model. Results are expressed as odds ratios (ORs) and 95% confidence intervals (CIs). Tests for trend were conducted by using likelihood ratio statistics in the conditional logistic regression models.

## RESULTS

The majority of women in the case–control study were Caucasian (82%), 11% were African American and 7% reported other ethnicities. This ethnic composition reflects the characteristics of the patient population in the screening clinic at the time of recruitment. Because of matching, the cases and controls had similar proportions of premenopausal and postmenopausal women (25 and 75%, respectively) at baseline and identical age at enrollment (mean 56 years, range 35–69 years). Common histopathological types of lung cancer were observed: 62 non-small cell types (37 adenocarcinomas, 16 bronchoalveolar adenocarcinomas, 4 squamous cell carcinomas, 3 large cell carcinomas, 2 adenosquamous carcinomas), 8 small cell carcinomas, 4 malignant carcinoid tumours, 1 sarcoma, and 6 tumours of unknown histology. Among the cases, the mean age at diagnosis was 64 years (range 39–78 years) and the mean period between date of enrollment and date of diagnosis was 8 years (range 0.2–14 years).

Selected characteristics of the cases and the controls are presented in Table 1

**Table 1:**
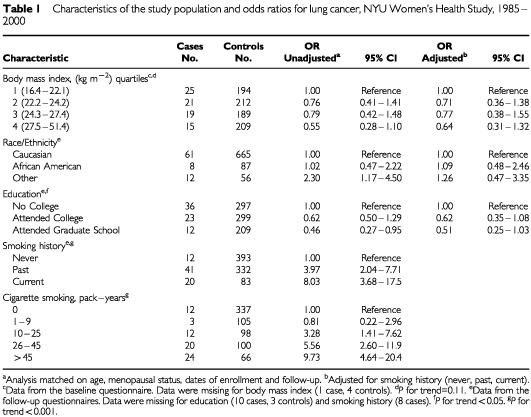
Characteristics of the study population and odds ratios for lung cancer, NYU Women's Health Study, 1985–2000

. Compared to never smokers, women who reported past smoking had a significantly higher risk of lung cancer (OR 3.97, 95% CI 2.04–7.71). The association was stronger among women who reported recent smoking during the follow-up period (OR 8.03, 95% CI 3.68–17.54). A significant positive trend was observed between the pack-years of cigarette smoking and risk of lung cancer (*P* for trend <0.001). In univariate analyses, body mass index and educational status were inversely associated with the risk of lung cancer (Table 1). However, after adjustment for smoking history the inverse association remained significant only for educational status (*P* for trend <0.05).

Exposure to aspirin in the 4 weeks preceding the enrollment did not appear to influence lung cancer risk (unadjusted OR 1.02, 95% CI 0.69–1.50). Adjustments for smoking and educational status did not change the risk estimate substantially (adjusted OR 0.93, 95% CI 0.56–1.53). Compared with nonusers, the odds ratio for lung cancer (all types) among women who reported aspirin use three or more times per week for a period of at least 6 months was 0.66 (95% CI 0.34–1.28) after adjustment for smoking and educational status (Table 2

**Table 2:**
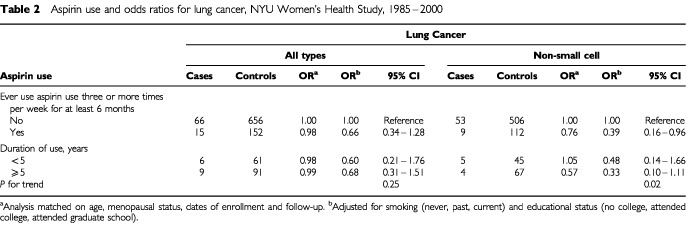
Aspirin use and odds ratios for lung cancer, NYU Women's Health Study, 1985–2000

).

Because previous studies have shown that increased cyclo-oxygenase-2 (COX-2) expression occurs preferentially in non-small cell lung cancers, and particularly, in adenocarcinomas and squamous cell carcinomas (Hida *et al*, 1998; Wolff *et al*, 1998; Ochiai *et al*, 1999), we analysed the association between aspirin use and lung cancer risk in a subset of 62 non-small cell lung cancer cases and 618 matching controls. Short-term aspirin use during the 4-week period prior to the enrollment was not associated with risk of non-small cell lung cancer (adjusted OR 0.96, 95% CI 0.56–1.63). Regular aspirin use for at least 6 months was inversely associated with risk of non-small cell lung cancer after controlling for smoking and educational status (adjusted OR 0.39, 95% CI 0.16–0.96). A significant inverse trend was observed between duration of aspirin use and risk of non-small lung cancer (*P* for trend=0.02) (Table 2).

Because 30 of the 81 cases reported on their long-term aspirin use after having been diagnosed with lung cancer, the above analyses were repeated after the exclusion of these cases and their matched controls. In these analyses (51 cases, 510 controls), regular aspirin use resulted in adjusted odds ratios of 0.54 (95% CI 0.23–1.24) for all types of lung cancer and 0.23 (95% CI 0.07–0.81) for non-small cell lung cancer, as compared with non-use.

## DISCUSSION

In a study of mostly Caucasian women in New York City, long-term aspirin use (for a period of at least 6 months) was inversely associated with the risk of lung cancer. The inverse association was particularly evident in relation to non-small cell lung cancer. After adjustment for smoking and educational status, the inverse association between long-term aspirin use and non-small cell lung cancer appeared to be stronger among women who reported aspirin use for a longer period of time (at least 5 years). Short-term aspirin use (during the 4-week period prior to enrollment) was not associated with risk of lung cancer (both all-types and non-small cell types).

Several methodological issues should be considered in the interpretation of the study results. The study limitations included the relatively small number of lung cancer cases, the dependence on a self-administered questionnaire for exposure assessment and lack of information on the indication, the dose and the precise duration of aspirin use. An additional concern was that information about long-term aspirin exposure was collected after the enrollment of study subjects in the cohort and, for a substantial proportion of the cases, after the occurrence of lung cancer, which raises the possibility of recall and survival biases. The recall bias may have been less likely because the exclusion of 30 cases who reported long-term aspirin use after the diagnosis did not affect the results significantly. Indeed, we would have expected recall bias to operate in the opposite direction to that observed, i.e., a tendency for cases to better recall an exposure would result in odds ratios greater than one rather than the inverse association observed here.

Survival bias is of concern for diseases characterised by poor prognosis, including lung cancer. Indeed, 63 cases (44%) had died before the mailing of the questionnaire eliciting information on long-term aspirin use and are, therefore, not included in the analyses of follow-up data. If aspirin use were associated with reduced survival following diagnosis, the proportion of aspirin users among case survivors would be lower than among controls, leading to an apparent inverse association between aspirin use and risk of lung cancer that would be spurious. The evidence on the effect of aspirin on survival of lung cancer patients is limited. However, several studies have shown that overexpression of COX-2, which is inhibited by aspirin, is associated with poor prognosis in lung cancer (Achiwa *et al*, 1999; Khuri *et al*, 2001), suggesting that if aspirin has an effect on survival in lung cancer, it is more likely to be beneficial than detrimental. In this case, the effect of survival bias would attenuate a real inverse association between aspirin use and lung cancer, suggesting that the protective effect of aspirin could in fact be larger than we observed.

To ensure that only relevant aspirin exposures were considered, we discounted exposures occurring after the diagnosis and within 1 year before diagnosis for each case and corresponding controls. An additional concern was the possibility of confounding by indication. Due to the lack of data on the reasons for aspirin use in our study, we were unable to separate the effects of aspirin from the indications for aspirin use. We also considered a possibility of exposure misclassification resulting from the inability to verify the use of cold remedies and other combined preparations containing acetylsalicylic acid, which may influence the observed association. However, one would expect such misclassification to be non-differential, with a tendency to obscure rather than establish associations.

The observed inverse association between long-term aspirin use and risk of lung cancer is in agreement with previous epidemiological studies suggesting that aspirin use could be associated with at least a moderately reduced risk of lung cancer (Peto *et al*, 1988; Schreinemachers and Everson, 1994). The observed association is also consistent with experimental evidence indicating that chemopreventive effects of aspirin and other NSAIDs could be mediated through the inhibition of COX-2 enzyme, which appears to be overexpressed in human lung carcinoma, particularly in non-small cell lung cancer (Hida *et al*, 1998; Wolff *et al*, 1998; Ochiai *et al*, 1999). In the lung, the interaction of inhaled particles with alveolar macrophages and structural epithelial cells leads to the inflammatory response characterised by activation of COX-2 enzyme, release of prostaglandins, leukotrienes, cytokines, reactive oxygen species, and growth factors that are involved in local tissue damage and remodelling (Janssen *et al*, 1993). The inflammatory response is crucial in the activation of proto-oncogenes by carcinogenic fibres (Janssen *et al*, 1994), mutations in the alveolar epithelial cells and subsequent development of lung neoplasia in animal models (Dungworth *et al*, 1994; Borm and Driscoll, 1996).

Growing epidemiological evidence indicates that inflammation may play an important role in lung cancer. Several studies have found that inflammatory lung diseases, including asthma and chronic bronchitis are associated with increased incidence of lung cancer (Alavanja *et al*, 1992; Kallen *et al*, 1993; Vesterinen *et al*, 1993; Wu *et al*, 1995; Huovinen *et al*, 1997; Mayne *et al*, 1999; Brownson and Alavanja, 2000; Brenner *et al*, 2001).

Aspirin and other NSAIDs share an ability to reduce inflammatory responses by inhibiting the synthesis of prostaglandins from arachidonic acid. Prostaglandins are important regulatory factors of epithelial cell function and, under certain circumstances, prostaglandins can stimulate cell proliferation and suppress immune response to neoplastic cells (Plescia *et al*, 1975). In addition, several groups have shown that high concentrations of free arachidonic acid can promote apoptosis, independently of prostaglandin formation (Surette *et al*, 1996; Chan *et al*, 1998; Longo *et al*, 1999). It has been proposed that the increased expression of COX-2 may lower the intracellular level of free arachidonic acid and thereby reduce apoptosis (Prescott and Fitzpatrick, 2000). Consequently, the chemopreventive effects of aspirin and other NSAIDs could result from inhibition of COX-2, reduction of prostaglandin synthesis, increased intracellular levels of free arachidonic acid, and induction of apoptosis.

In conclusion, the results of the study suggest that regular aspirin use may be inversely associated with lung cancer in women, indicating that aspirin may have broader chemopreventive properties than previously thought. Because regular aspirin use may occasionally result in serious side effects, such as gastrointestinal bleeding, hemorrhagic stroke, and aspirin-sensitive asthma, specific recommendations regarding use of aspirin for prevention of lung cancer should be deferred until confirmation of the effect by larger studies and determination of the effective dose and duration of use.
